# Liraglutide pharmacokinetics and exposure‐response in adolescents with obesity

**DOI:** 10.1111/ijpo.12799

**Published:** 2021-05-07

**Authors:** Kristin C. Carlsson Petri, Paula M. Hale, Dan Hesse, Naveen Rathor, Lucy D. Mastrandrea

**Affiliations:** ^1^ Department of Quantitative Clinical Pharmacology Novo Nordisk A/S Søborg Denmark; ^2^ Clinical Development, Medical & Regulatory Affairs Novo Nordisk Inc Plainsboro New Jersey USA; ^3^ Department of Medical & Science – Obesity and Metabolism Novo Nordisk A/S Søborg Denmark; ^4^ Department of Global Medical Affairs Novo Nordisk A/S Søborg Denmark; ^5^ Division of Pediatric Endocrinology/Diabetes, Jacobs School of Medicine and Biomedical Sciences University at Buffalo Buffalo New York USA

**Keywords:** adolescents, clinical trial, GLP‐1, liraglutide, paediatric, pharmacokinetics

## Abstract

**Background:**

Obesity in adolescence presents a major public health challenge, often leading to obesity in adulthood with associated chronic disease.

**Objectives:**

This study aimed to perform a population pharmacokinetic and exposure‐response analysis of liraglutide by meta‐analysis of data from trials conducted in children, adolescents and adults with obesity.

**Methods:**

The population pharmacokinetic analysis investigated the effect of covariates body weight, age group (children, adolescents and adults) and sex on liraglutide exposure in adolescents compared with previous results in adults. The exposure‐response relationship of liraglutide for the change from baseline in body mass index standard deviation score (BMI SDS) was evaluated in adolescents and compared to that in adults.

**Results:**

Body weight was the main covariate affecting liraglutide exposure, with lower exposures at higher body weights, whereas age group was of no importance and sex was of little importance. An exposure‐response relationship was demonstrated for liraglutide in both adolescents and adults as the decrease in BMI SDS from baseline increased in an exposure‐dependent manner with increasing liraglutide exposure.

**Conclusions:**

The population pharmacokinetic analysis supported similar liraglutide exposures in adolescents and adults; body weight was the most important covariate affecting exposure. An exposure‐response relationship was established for liraglutide.

## 1 INTRODUCTION

The prevalence of obesity over the past 30 years has more than doubled in children and tripled in adolescents, reaching epidemic proportions in the United States.^1^, ^2^ While the overall prevalence of obesity is lower in children than in adults, rates of increase in childhood obesity are now higher than those seen in adults in many countries.^3^ Childhood obesity presents a major public health challenge since obesity in childhood and adolescence often leads to obesity in adulthood.^4^, ^5^, ^6^ Paediatric obesity is associated with a wide range of chronic diseases, including type 2 diabetes, hypertension, hyperlipidaemia, polycystic ovary syndrome and sleep apnea.^7^, ^8^, ^9^, ^10^ Prolonged obesity continuing into adulthood may lead to complications such as cardiovascular disease,^11^, ^12^ which was responsible for more than two‐thirds of adult deaths associated with high body mass index (BMI) worldwide in 2015.^3^ Childhood and adolescent obesity may also adversely affect quality of life, resulting in adverse psychosocial problems such as low self‐esteem, depression and reduced educational achievement.^4^, ^8^ The economic consequences of obesity and its related complications are also high.^13^, ^14^

Current treatments for children and adolescents with obesity tend to favour lifestyle modifications that target diet and exercise; however, such programs often have limited effect on reducing BMI in adolescents because weight loss and maintenance of weight loss are hard to achieve.^15^, ^16^ Bariatric surgery may be an effective alternative for individuals with morbid obesity,^16^, ^17^ though there remains a treatment gap for patients who do not meet criteria for surgery and those who struggle to lose weight with lifestyle interventions alone. Few approved pharmacotherapies are available for the treatment of obesity in the paediatric population. In the United States, liraglutide 3.0 mg was recently approved for chronic weight management by the Food and Drug Administration (FDA) for use in patients aged ≥12 years weighing >60 kg. Both orlistat (patients ≥12 years) and phentermine (patients >16 years) are also approved for weight management, and setmelanotide is approved for the treatment of certain genetic causes of obesity (patients ≥6 years). Liraglutide 3.0 mg is also approved for use in adolescents in Brazil and Saudi Arabia and orlistat is approved in Switzerland for adolescent use by the Swiss Regulatory Agency, Swissmedic.

Glucagon‐like peptide‐1 (GLP‐1), an incretin hormone secreted by intestinal L cells in response to food intake,^18^ stimulates insulin secretion and inhibits glucagon secretion in a glucose‐dependent manner.^18^, ^19^ Liraglutide is a GLP‐1 receptor agonist that promotes weight loss through reduced appetite and a subsequent reduction in energy intake.^20^ As an adjunct to a reduced‐calorie diet and increased physical activity, liraglutide 3.0 mg (Saxenda) once daily is approved in the United States, European Union and elsewhere (65 countries) for chronic weight management in adults with obesity (BMI ≥30 kg/m^2^), or overweight (≥27 to <30 km/m^2^) in the presence of at least one weight‐related comorbidity.

The efficacy and safety of liraglutide in adults were evaluated in the Satiety and Clinical Adiposity – Liraglutide Evidence (SCALE) program. The safety, tolerability, pharmacokinetics and pharmacodynamics of liraglutide in adolescents aged 12‐17 years^21^ and in children aged 7‐11 years^22^ have previously been investigated in short‐term (<10 weeks) randomized, controlled trials. In phase 3 randomized, controlled 56‐week trial investigating the effects of liraglutide for weight management in pubertal adolescents with obesity, liraglutide, in addition to lifestyle therapy, significantly reduced the BMI standard‐deviation score (SDS) compared with placebo (estimated treatment difference: −0.22; 95% confidence interval [CI], −0.37, −0.08; *P* = 0.002).^23^

A previous population pharmacokinetic analysis performed with liraglutide using adult data from the 56‐week phase 3a SCALE Obesity and Prediabetes trial (1839)^24^ and SCALE Diabetes trial (1922)^25^ trials found that sex and body weight were the only intrinsic factors that influenced the exposure (ie, the average steady‐state plasma concentration) of liraglutide 3.0 mg.^26^ Moreover, in a previous exposure‐response analysis using data from the aforementioned SCALE trials and a phase 2, 20‐week trial (1807),^27^ greater weight loss was observed with higher liraglutide exposures.^28^ This report aimed to perform population pharmacokinetic and exposure‐response analyses of data from the recent 56‐week trial in adolescents (trial 4180) in a meta‐analysis including data from previous trials conducted in children, adolescents and adults with obesity, to support the 3.0 mg liraglutide dose for weight management in an adolescent population. The population pharmacokinetic analysis will provide additional information including adolescent data from a long‐term (52‐week) phase 3 trial, to support previous findings. The exposure‐response analysis is the first to evaluate phase 3 data from both adults and adolescents together.

## 2 METHODS

### 2.1 Population pharmacokinetic analysis

The objectives of the population pharmacokinetic analysis were (1) to investigate body weight, age group (children aged 7‐11 years, adolescents aged 12‐17 years and adults aged ≥18 years) and sex as covariates to determine whether their impact on liraglutide exposure was in accordance with previous results in adults^26^ and (2) to compare the drug exposure for adolescents in the 56‐week trial 4180^23^ to previous results from shorter trials in adolescents, children and adults. As the focus of the analysis was a comparison of different age populations, age was prespecified to be included initially as a categorical variable. If proven to be an important covariate, further exploration of this relationship was possible if relevant; however, this was not the case.

Data from trials 4180 and 3967 (in adolescents), 4181 (children) and 3630 (adults) were included in the pharmacokinetic assessment of liraglutide (Table 1). In trial 4180, Tanner staging (stages 2‐5) of adolescents was assessed by site staff trained in pubertal assessments. For females, assessments of breast and pubic hair development were made. For males, assessments of testicular volume (by orchidometer), penis development and pubic hair development were made. Prepubertal patients were not permitted in the trial. In all trials, liraglutide concentration in plasma was determined using a validated enzyme‐linked immuno‐sorbent assay (ELISA) with a lower limit of quantification (LLOQ) of 0.03 nmol/L.^29^ The ELISA was a sandwich immunoassay with two monoclonal antibodies directed against different epitopes on liraglutide and was according to guidance regarding recovery, accuracy, precision, sensitivity and stability.

**TABLE 1:** Trial design and baseline characteristics of the trials included in the population PK analysis

	Trial 4180^23^ (NCT02918279) Phase 3a trial in adolescents N = 121	Trial 4181^22^ (NCT02696148) Phase 1 trial in children N = 13	Trial 3967^21^ (NCT01789086) Phase 1 trial in adolescents N = 13	Trial 3630^20^ (NCT00978393) Phase 1 trial in adults N = 29
*Trial design*				
Weekly dose escalation steps (mg/day)	0.6, 1.2, 1.8, 2.4, 3.0	0.3, 0.6, 0.9, 1.2, 1.8, 2.4, 3.0	0.6, 1.2, 1.8, 2.4, 3.0	0.6, 1.2, 1.8, 2.4, 3.0
Maintenance doses (mg/day)	0.6 (n = 1); 1.2 (n = 1); 1.8 (n = 2); 2.4 (n = 10); 3.0 (n = 107)	2.4 (n = 1); 3.0 (n = 12)	2.4 (n = 1); 3.0 (n = 12)	3.0
Treatment duration^a^	56 weeks	7 weeks	5‐6 weeks	35 days
Completed trial (% liraglutide vs placebo)	81%; 79%	88%; 75%	93%; 100%	90% across groups
Sparse PK sampling weeks	8, 12, 16, 30, 42, 56	NA	NA	NA
Number of pre‐dose (trough) PK samples during dose escalation	NA	7^b^	4	NA
Number of PK samples after last dose	NA	5	6	12
Nominal timing of PK sampling after the last dose	NA	Pre‐dose, 1, 2, 3, 24, 72 hours	Varying according to assigned sequence	Pre‐dose, 2, 4, 1, 13, 15, 18, 20, 24, 36, 48, 60 hours
*Demographics*				
Sex				
Female	67 (55.4%)	7 (53.8%)	10 (76.9%)	11 (37.9%)
Male	54 (44.6%)	6 (46.2%)	3 (23.1%)	18 (62.1%)
Race				
White	102 (84.3%)	6 (46.2%)	12 (92.3%)	25 (86.2%)
Black or African American	13 (10.7%)	7 (53.8%)	–	–
Asian	2 (1.7%)	–	–	–
Other	4 (3.3%)	‐	1 (7.7%)	4 (13.8%)
Ethnicity				
Not Hispanic or Latino	92 (76.0%)	10 (76.9%)	13 (100%)	26 (89.7%)
Hispanic or Latino	29 (24.0%)	3 (23.1%)	–	3 (10.3%)
Age, years (mean [SD])	14.6 (1.6)	9.8 (0.9)	15.1 (1)	47.8 (13.8)
Range	12–17	8‐11	13‐16	20‐72
Body weight, kg (mean [SD])	99.4 (19.7)	69.1 (10.8)	102.1 (12.2)	102.3 (15.6)
Range	62.1‐178.2	53.9‐86.8	79.9‐119.2	74.2‐131.6

*Note*: Data for demographics are presented as number and percentage of participants, unless otherwise stated.

Abbreviations: N, number of participants in the population PK analysis (those on liraglutide); NA, not applicable; PK, pharmacokinetic; SD, standard deviation.

^a^ Including dose‐escalation.

^b^Including one trough sample before last dose.

A standard one‐compartment model with first‐order absorption and elimination was the starting point for the description of liraglutide pharmacokinetics, and the model was developed and validated according to the US FDA and European Medicines Agency guidelines.^30^, ^31^ The structural model was parameterized in terms of the following parameters:

- k_a_ (absorption rate constant)
- CL/F (apparent clearance)
- V/F (apparent volume of distribution)

The first‐order conditional estimation with interaction (FOCE‐I) method was used for the population pharmacokinetic analysis, implemented in the non‐linear mixed effects modelling (NONMEM) software. Due to pharmacokinetic sampling in steady‐state conditions, exposure levels were expected to be much higher than the LLOQ (mean values 500‐ to 1000‐fold above LLOQ). Observed values close to or below the LLOQ were therefore believed to be due to missed doses. Data records with missing concentration values or concentration values below the LLOQ were excluded from the population pharmacokinetic analysis in order not to overestimate CL/F. Stricter exclusion criteria were tested in sensitivity analyses but were found not to be relevant.

An analysis of the influence of covariates on exposure was carried out including all tested covariates in one step, using a confirmatory approach,^32^ and disregarding any interactions between age group and sex. Model development involved estimation of a base model without covariates and a full model including all predefined covariates. If any covariates without statistically significant effect could be excluded, the reduced model would be considered the final population pharmacokinetic model. The covariates were prespecified and based on earlier findings in phase 3 trials with adults as well as prior knowledge from smaller trials in children and adolescents.

The covariate effects of baseline body weight, sex and age group were investigated for CL/F. Due to the sparseness of data, as well as the focus on average exposure, only the effect of baseline body weight was investigated with respect to V/F. The CL/F and V/F were parameterized as follows for the i^th^ subject (shown for CL/F only):

(1) $$\mathrm{CL}/F_{i}=\text{TVCL}∙E_{\text{body weight},\mathrm{CL}}∙E_{\mathrm{sex}}∙E_{\mathrm{age}\;\text{group}}∙\exp\left(\eta_{\mathrm{CL},i}\right)\;$$

(2) $$E_{\text{body weight},\mathrm{CL}}={\left(\frac{{\text{body weight}}_{i}}{100\;\mathrm{kg}}\right)}^{\theta_{\text{body weight},\mathrm{CL}}}$$

(3) $$E_{\mathrm{sex}}={\left(\theta_{\text{male}}\right)}^{\text{male}}$$

(4) $$E_{\mathrm{age}\;\text{group}}={\left(\theta_{\text{child}}\right)}^{\text{child}}∙{\left(\theta_{\text{adolescent}}\right)}^{\text{adolescent}}$$

(5) $$V/F_{i}=\mathrm{TVV}∙E_{\text{body weight},V}∙\exp\left(\eta_{V,i}\right)$$

where TVCL and TVV are “typical values (TV)” of apparent clearance (CL/F) and volume of distribution (V/F), respectively, for a reference subject (an adult female with a body weight of 100 kg) and the θ values are the estimated covariate effect parameters.

Specific steady‐state exposures for individual participants were based on the full model, including all covariates, and derived from the subject‐specific post‐hoc CL/F estimates, the maintenance dose and the dose interval (24 hours):

(6) $$C_{\mathrm{avg}}=\frac{\text{Dose}}{\left(\frac{\mathrm{CL}}{F}\right)*24\;\text{hours}}$$

The CIs for full model parameters were estimated using bootstrapping. For each investigated covariate, differences were considered relevant if the 90% CI of the estimated mean of the relative exposure fell outside the standard bioequivalence limits (0.80‐1.25).

Between‐subject variability was included for CL/F and V/F, assuming log‐normal distributions without correlation between parameters. Furthermore, CL/F and V/F were estimated using a full variance‐covariance matrix. No between‐subject variability was included for k_a_. Within‐subject variability (residual) was described by a proportional error model. Standard graphical quality analyses, including goodness‐of‐fit plots (Figure S1), were made during qualification of the pharmacokinetic model.

### 2.2 Exposure‐response analysis

The objectives of the exposure‐response analysis were: (1) to investigate the exposure‐response relationship of liraglutide in adolescents with respect to the change from baseline in BMI SDS and (2) to determine whether the exposure‐response relationship for the change from baseline in BMI SDS was similar in adults and adolescents.

An evaluation of the exposure‐response of liraglutide in adults has been published previously using data from the phase 2 trial (1807), the phase 3a SCALE Obesity and Prediabetes trial (1839) and the phase 3a SCALE Diabetes trial (1922).^28^ Data from the phase 3a trial 4180 (in adolescents) were additionally included in the present exposure‐response assessment of liraglutide (Table 2). The analyses were conducted using response data at week 20 (phase 2 trial 1807), week 50 (phase 3a trial 1922) or week 56 (phase 3a trials 4180 and 1839) to align with the original analysis in adults. The appropriateness of using different trial lengths for the historical adult trials was evaluated by graphically exploring the weight loss longitudinally. This was deemed appropriate as the maximal effect on weight reduction was reached after 20 weeks of treatment and remained stable thereafter for the trials of longer duration.

**TABLE 2:** Trial design and baseline characteristics of the trials included in the exposure‐response analysis

	Trial 4180^23^ (NCT02918279) Phase 3a trial in adolescents N = 247	Trial 1807^27^ (NCT00422058) Phase 2 trial in adults N = 415	Trial 1839^24^ (NCT01272219) Phase 3a trial in adults N = 3250	Trial 1922^25^ (NCT01272232) Phase 3a trial in adults N = 707
*Trial design*				
Participants on liraglutide treatment	121	331	2339	584
Participants on placebo treatment	126	84	911	123
Participants with normoglycemia	183	205	1250	0
Participants with prediabetes	62	210	2000	0
Participants with type 2 diabetes	2	0	0	707
Weekly dose escalation steps (mg/day)	0.6, 1.2, 1.8, 2.4, 3.0	0.6, 1.2, 1.8, 2.4, 3.0	0.6, 1.2, 1.8, 2.4, 3.0	0.6, 1.2, 1.8, 2.4, 3.0
Maintenance doses (mg/day)	0.6 (n = 1); 1.2 (n = 1); 1.8 (n = 2); 2.4 (n = 10); 3.0 (n = 107)	1.2, 1.8, 2.4, 3.0	3.0	1.8, 3.0
Treatment duration^a^	56 weeks	20 weeks	56 weeks	56 weeks
Completed trial (% liraglutide vs placebo)	81%; 79%	85%; 81%	72%; 64%	77%; 66%
*Demographics*				
Sex				
Female	145 (58.7%)	313 (75.4%)	2535 (78%)	352 (49.8%)
Male	102 (41.3%)	102 (24.6%)	715 (22%)	355 (50.2%)
Race				
White	217 (87.9%)	413 (99.5%)	3072 (94.5%)	669 (94.6%)
Asian	2 (0.8%)	–	118 (3.6%)	16 (2.3%)
Black or African American	19 (7.7%)	–	‐	‐
American Indian or Alaska Native	1 (0.4%)	‐	7 (0.2%)	4 (0.6%)
Native Hawaiian or other Pacific Islander	–	‐	2 (0.1%)	–
Other	8 (3.2%)	2 (0.5%)	51 (1.6%)	18 (2.5%)
Ethnicity				
Not Hispanic or Latino	194 (78.5%)	415 (100%)	2913 (89.6%)	638 (90.2%)
Hispanic or Latino	53 (21.5%)	‐	337 (10.4%)	69 (9.8%)
Age, years (mean [SD])	14.5 (1.6)	46.4 (10.4)	45.3 (11.9)	54.8 (10.2)
Range	12–17	18‐65	18‐78	24‐82
Body weight, kg (mean [SD])	100.8 (20.7)	97.7 (12.9)	106.7 (21.4)	106.0 (21.2)
Range	62.1–178.2	69.2‐141.2	63.0‐244.0	60.1‐193.3
BMI, kg/m^2^ (mean [SD])	35.6 (5.4)	34.4 (2.8)	38.4 (6.3)	37.2 (6.8)
Range	26.6‐58.8	29.1‐41.0	27.0‐77.2	27.0‐67.6
BMI SDS^b^ (mean [SD])	3.2 (0.7)	2.8 (0.4)	3.4 (1.0)	3.2 (1.1)
Range	2.1‐6.5	1.9‐3.7	1.4‐9.3	1.4‐8.4

*Note*: Data for demographics are presented as number and percentage of participants, unless otherwise stated.

Abbreviations: BMI, body mass index; BMI SDS, body mass index standard deviation score; N, number of participants in the exposure‐response analysis; NA, not applicable; PK, pharmacokinetic; SD, standard deviation.

^a^ Including dose‐escalation.

^b^ BMI SDS represents the number of SDs from a reference standard population mean BMI.

The exposure variable used for the analysis was individual model‐based average liraglutide concentration (C_avg_) estimates from the population pharmacokinetic analyses of the four trials (for trial 4180, LLOQ samples were included in the exposure‐response data set to better reflect actual exposure ‐ accounting for potential non‐compliance). For the primary exposure‐response analysis of change from baseline in BMI SDS, BMI SDS was calculated according to the World Health Organization (WHO).^33^ For adults, the growth charts for 19‐year‐old females or males were used for the calculation. The following response variables were evaluated as supportive analyses: changes from baseline in body weight (%), BMI (%), waist circumference (cm) and the proportion of participants achieving a 5% reduction in body weight.

A modified exposure‐response model based on the previous meta‐analysis^28^ was used in the development of the present exposure‐response model. The starting models included the effect of all investigated covariates on the placebo effect E_0_, and of selected covariates on the E_max_, as identified based on previous analyses.^28^ The covariate selection was made as follows. Covariates for E_0_ were removed in a stepwise manner, via backwards elimination based on a likelihood ratio test, keeping the covariates that were both statistically significant (*P* < 0.05) and clear (ie, clearly needed to describe a potential difference in observed exposure‐response when stratified by the covariate). Based on the reduced model from the previous step, covariates for E_max_ were investigated via forwards inclusion. Based on plots of exposure‐response stratified by covariates, each covariate factor was considered. If the data indicated differences in the treatment effect, the covariates were investigated by forwards inclusion and included if statistically significant (*P* < 0.05). If the covariates appeared to be relevant based on the plots, they were tested in the following order: predefined covariates for E_max_ were investigated first, then other clear covariates for E_max_.

Standard goodness‐of‐fit plots were made for checking and evaluating the model.

The final models were parameterized as follows:

(7) $$\mathrm{BMI}\;{\mathrm{SDS}}_{\mathrm{CFB}}=E_{0}+E_{\max}\frac{C_{\mathrm{avg}}}{C_{\mathrm{avg}}+{\mathrm{EC}}_{50}}+E_{\mathrm{cov}}+e,$$

where CFB denoted the “change from baseline” in BMI SDS, E_0_ was the placebo effect, E_max_ was the maximal drug effect, EC_50_ the exposure leading to half‐maximum effect, E_cov_ covariate effects on the overall response (ie, placebo and treatment‐related responses) and *e* the normal distributed residual error. Sex, baseline response parameter, age group and trial factors were considered as covariates. Baseline BMI SDS and age group were included as covariates in the final model.

The exposure‐response evaluation for changes from baseline in body weight, BMI and waist circumference were evaluated using similar models as for the BMI SDS:

(8) $${\text{Body weight}}_{\mathrm{CFB}}=E_{0}+E_{\max}\frac{\left(1+{\left(I_{\text{adolescent}}\right)}^{\text{adolescent}}+{\left(I_{\text{male}}\right)}^{\text{male}}\right)∙{C_{\mathrm{avg}}}^{\gamma }}{{C_{\mathrm{avg}}}^{\gamma }+{{\mathrm{EC}}_{50}}^{\gamma }}+E_{\mathrm{cov}}+e$$

(9) $${\mathrm{BMI}}_{\mathrm{CFB}}=E_{0}+E_{\max}\frac{\left(1+{\left(I_{\text{male}}\right)}^{\text{male}}\right)∙{C_{\mathrm{avg}}}^{\gamma }}{{C_{\mathrm{avg}}}^{\gamma }+{{\mathrm{EC}}_{50}}^{\gamma }}+E_{\mathrm{cov}}+e$$

(10) $${\text{Waist}}_{\mathrm{CFB}}=E_{0}+E_{\max}\frac{C_{\mathrm{avg}}}{C_{\mathrm{avg}}+{\mathrm{EC}}_{50}}+E_{\mathrm{cov}}+e$$

I_adolescent_ was the covariate representing a lower E_max_ in adolescents compared to adults, where adolescent was an indicator variable for adolescents. I_male_ was the covariate representing a lower E_max_ in males compared to females, where *male* was an indicator variable for males. ɣ was the Hill coefficient. In the final models, E_cov_ covariate effects for body weight and BMI included age group, while for waist circumference the covariate effects were baseline waist circumference (cm), age group and diabetic state (ie, diabetes vs non‐diabetes). The “non‐diabetes” also included individuals with prediabetes. The other expressions were as defined above.

The exposure‐response relationship for the proportion of participants with at least 5% reduction in body weight (body weight responder rate [BW.RR]) was estimated using logistic regression analyses parameterized as:

(11) $$\mathrm{BW}.\mathrm{RR}∼\text{invlogit}\left(\frac{E_{\max}\;\left(1+I_{\text{male}}\right)∙{c_{\mathrm{avg}}}^{\gamma }}{{C_{\mathrm{avg}}}^{\gamma }+{{\mathrm{EC}}_{50}}^{\gamma }}+E_{0}+E_{\mathrm{cov}}\;\right)$$

The expressions were as defined above. In the final model, age group was included as a covariate effect.

### 2.3 Data analysis software

The software program NONMEM (ICON Development Solutions, Ellicott City, MD, USA) version 7.3 was used for the population pharmacokinetic analysis. R version 3.2.3 (R Foundation, Revolution Analytics, Mountain View, CA, USA) was used for data file processing, explorative data analysis and plotting. Exposure‐response analyses were implemented in R.

## 3 RESULTS

### 3.1 Population pharmacokinetic analysis

#### 3.1.1 Model development and qualification

A base model without covariates was developed based on prior knowledge of liraglutide pharmacokinetics. Subsequently, a full model, which was the base model with all investigated covariates included, was estimated for the covariate analysis. All investigated covariates were significant, except for age group. The full model could therefore not be reduced and, therefore, the full model was adopted as the final model.

A one‐compartment model successfully described the pharmacokinetics of liraglutide. The parameter estimates from the base and final models are presented in Table S1 and Table 3, respectively.

**TABLE 3:** Parameter estimates from the final pharmacokinetic model

Parameter	Parameter symbol (unit)	Estimate	95% CI lower bound	95% CI upper bound	RSE (%)	IIV (%CV)	Shrinkage (%)
Absorption rate constant	KA (1/h)	0.0813	Fixed	Fixed	Fixed	NA	NA
Apparent clearance	CL/F (L/h)	1.01	0.922	1.09	4.25	31.2	10.2
Apparent volume of distribution	V/F (L)	13.8	Fixed	Fixed	Fixed	31.7	19.2
Body weight exponent on CL/F	CL‐BW	0.762	0.565	0.958	13.2	NA	NA
Sex contrast (male/female) on CL/F	CL‐Male	1.12	0.993	1.24	5.64	NA	NA
Age contrast (child/adult) on CL/F	CL‐Child	1.11	0.89	1.34	10.2	NA	NA
Age contrast (adolescent/adult) on CL/F	CL‐Adole	1.06	0.931	1.19	6.24	NA	NA
Body weight exponent on V/F	V‐BW	0.587	0.475	0.7	9.75	NA	NA
NA	Prop. Error	43.3	NA	NA	NA	NA	6.4

Abbreviations: CI, confidence interval; CV, coefficient of variation; RSE, relative standard error.

#### 3.1.2 Demographics and datasets

A total of 176 individuals was included in the analysis: 13 children from trial 4181, 121 adolescents from trial 4180, 13 adolescents from trial 3967 and 29 adults from trial 3630 (Table 1). The majority of participants (82.4%) were white (11.4% were black or African American) and non‐Hispanic (80.1%).

For trial 4180, the final dataset comprised 646 pharmacokinetic observations from 121 adolescents (94 observations [14.6% of the final dataset] were below the LLOQ); 22 samples from two individuals were excluded during data cleaning due to timing being missing or an inadequate dosing history. There were also many lower‐than‐expected individual liraglutide values in trial 4180 (Figure S2). Data cleaning for trials 4181, 3967 and 3630 was conducted similarly to previously reported analyses.^21^, ^22^ The number of observations included for each trial as well as the number of samples below the LLOQ and excluded is presented by trial in the Supporting Information.

#### 3.1.3 Covariate analysis

**FIGURE 1:**
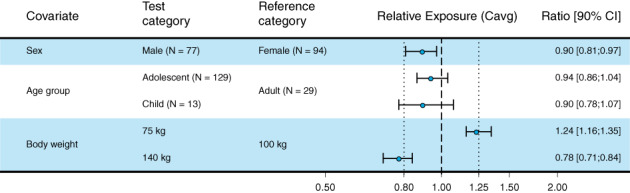
Effect of covariates on liraglutide exposure in adults, adolescents and children with obesity. The reference category profile was a female adult with a body weight of 100 kg. The body weight test categories (75 and 140 kg) represent the approximate 5% and 95% percentiles in the combined data set. The column to the right shows numerical means and 90% CI for the relative exposures. Vertical dotted lines indicate the acceptance interval for bioequivalence (0.80‐1.25). Data are included from trials 4180, 3967, 3630 and 4181. C_avg_, average liraglutide concentration; CI, confidence interval; N, number of participants

The effects of intrinsic covariates on liraglutide exposure are shown in Figure 1. All covariates were tested simultaneously; thus for a given covariate effect, the effects of the other covariates were accounted for. In accordance with previous findings in adults, body weight was the main intrinsic covariate affecting liraglutide exposure, with lower exposure at higher body weights. Age group was of no importance and sex was of little importance.

**FIGURE 2:**
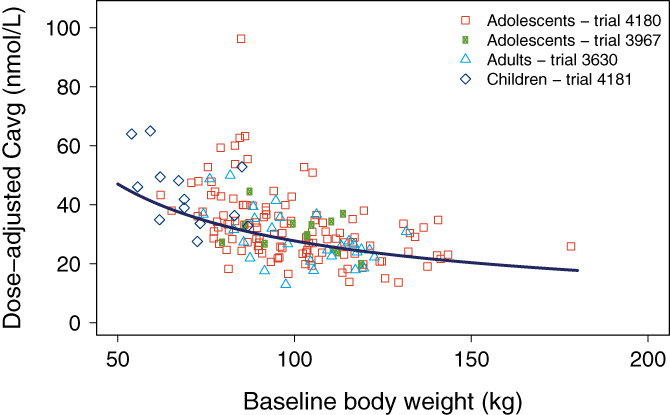
Liraglutide exposure vs baseline body weight. Data are individual values of C_avg_ vs baseline body weight (symbols). The line represents the mean covariate‐adjusted model‐derived concentrations vs body weight for 3.0 mg dose across all age groups. Data are included from trials 4180, 3967, 3630 and 4181. C_avg_, average liraglutide concentration

**FIGURE 3:**
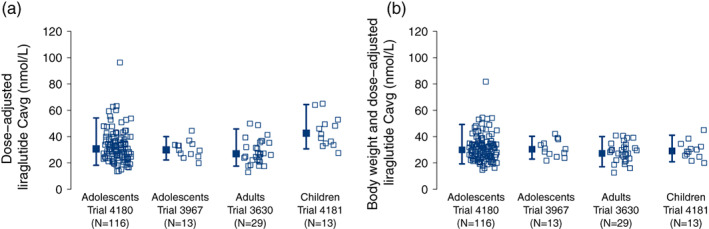
Liraglutide steady‐state exposure across trials without (A) and with (B) adjustment for body weight. Data are individual (open symbols) and geometric mean C_avg_ estimates adjusted to the liraglutide 3.0 mg dose with 90% range (closed symbols with error bars) from the final PK model for each trial. Data are included from trials 4180, 3967, 3630 and 4181. C_avg_, average liraglutide concentration; N, number of participants; PK, pharmacokinetic

The inverse relationship between body weight and exposure was apparent across trials and age groups (Figure 2). Individual and mean C_avg_ values appeared to be similar in adolescents and adults when adjusted to the 3.0 mg dose, whereas children had slightly higher liraglutide concentrations (Figure 3A). When adjusted for differences in body weight, however, exposures were similar across all age groups (Figure 3B).

### 3.2 Exposure‐response analysis

#### 3.2.1 Demographics and datasets

In the exposure‐response full dataset, there were more females than males in most of the trials, except for trial 1922 in which sex was evenly distributed (Table 2). Otherwise the demographic characteristics were generally similar across trials. Several race and ethnicity categories were represented for both adolescents and adults, with 7.7% of participants black or African American in trial 4180 (Table 2). The mean baseline body weight was 100.8 kg in adolescents from trial 4180 and ranged from 97.7 kg in the phase 2 trial in adults (trial 1807) to 106.7 kg in the largest phase 3 trial in adults (trial 1839). Baseline body weights and BMI SDS values were similar between adolescents and adults (Figure S3).

The exposure‐response dataset comprised placebo and treatment data from a total of 4619 individuals: 4372 adults from trials 1807, 1839 and 1922 and 247 adolescents from trial 4180. Last‐observation‐carried‐forward (LOCF) imputation was used if the observation was missing at the end of treatment, consistent with the original analysis.^28^ Data for waist circumference were not obtained for all adult trial participants, so this dataset included a total of 3163 individuals.

#### 3.2.2 Liraglutide exposure‐response relationship in adolescents and adults

**FIGURE 4:**
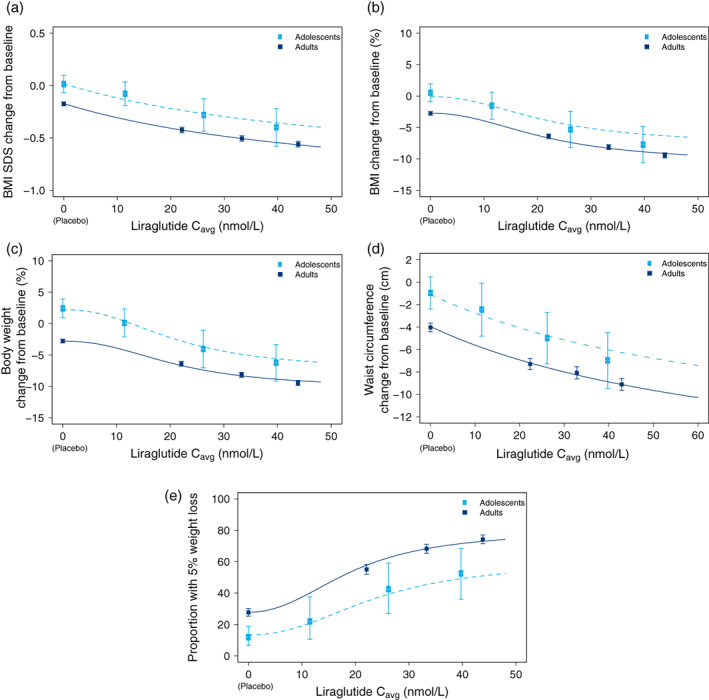
Liraglutide exposure‐response relationships in adults and adolescents. Figures show the weight‐related outcomes for liraglutide exposure in adolescents and adults with respect to A: BMI SDS, B: BMI (%), C: body weight (%), D: waist circumference (cm) and E: the proportion of individuals achieving 5% weight reduction. Data points with error bars are arithmetic means with 95% CIs for each of 3 quantiles of C_avg_ for liraglutide and one quantile for placebo (at C_avg_ of 0 nmol/L). Lines are covariate‐adjusted, model derived relationships. Data are included from trial 1807 after 20 weeks of treatment, trials 4180 and 1839 after 56 weeks of treatment and trial 1922 after 50 weeks of treatment. BMI, body mass index, BMI SDS, body mass index SD score; C_avg_, average liraglutide concentration; CI, confidence interval; N, number of participants; PK, pharmacokinetic

An exposure‐response relationship was demonstrated for liraglutide in both adolescents and adults with a greater decrease from baseline in BMI SDS as liraglutide exposure increased (Figure 4A). Similarly, an exposure‐response relationship, with larger responses at higher exposures, was identified for changes from baseline in BMI (%) (Figure 4B), body weight (%) (Figure 4C), waist circumference (Figure 4D) as well as the proportion of participants achieving at least a 5% weight loss (Figure 4E).

For all response variables, similar exposure‐response relationships were seen in both adolescents and adults. The two age groups differed in the placebo group response, with lower mean responses in adolescents; this difference was consistent across the exposure range as well. There was a tendency for a steeper exposure‐response in adolescents for all response variables, leading to overlapping effects with adults at the highest liraglutide exposures. However, the effect of age group on the maximal effect in the model (E_max_) was not significant in these analyses. The exposure‐response for adolescents in trial 4180 who gained height of at least 0.5 cm during the trial was not significantly different from the exposure‐response for those who had reached their final height. Approximately 64% of the adolescents in the exposure‐response analysis grew more than 0.5 cm during the trial. Of these, some received liraglutide and some received placebo. For those receiving placebo treatment, the average growth was 1.8 cm, while for those receiving liraglutide treatment, the average growth was 1.4 cm (data not shown).

In accordance with the similar demographic characteristics of adults and adolescents included in the exposure‐response analyses, the exposures of liraglutide were overlapping in the two populations, as shown for all participants with exposures adjusted to the liraglutide 3.0 mg dose (Figure S4). In trial 4180, liraglutide exposures were also similar in adolescents in earlier stages of pubertal development (Tanner stages 2 and 3) as compared with those in later stages (Tanner stages 4 and 5) (Figure S5).

## 4 DISCUSSION

The present population pharmacokinetic analysis demonstrates that the principal covariate affecting the exposure of liraglutide 3.0 mg is body weight, with lower exposures as body weight increases. Age group was not an important covariate and, as the body weight ranges were similar between adolescent trial participants and historical adult trial participants, the exposure ranges in the two populations were comparable. An exposure‐response relationship was demonstrated for liraglutide in both adolescents and adults, with larger responses at higher exposures. Thus, as liraglutide exposures increased, the mean changes from baseline in BMI SDS, BMI (%), body weight (%), waist circumference and the proportion of participants achieving at least a 5% weight loss increased in an exposure‐dependent manner.

The recommended 3.0 mg liraglutide dose was shown to provide similar exposures in adolescents and adults, even without adjusting for baseline body weight, which supports using the same dose as approved in adults for weight management in adolescents. The added benefits of increasing liraglutide exposures, in terms of a greater reduction in BMI SDS and greater changes in other response variables, were similar in adolescents with obesity and adults with overweight or obesity, thus further supporting the 3.0 mg treatment dose. While individual and mean exposures appeared to be slightly higher in children than in adolescents or adults, exposures were similar across age groups after adjusting for body weight.

Although body weight was found to be the most important covariate affecting exposure and an exposure‐response relationship was demonstrated, the maintenance dose for liraglutide 3.0 mg is not dose‐adjusted according to body weight.^34^, ^35^ All phase 3 trials in the liraglutide development program evaluated a 3.0 mg maintenance dose in both adults and paediatric patients without weight‐adjusted dosing.^23^, ^24^, ^25^, ^36^, ^37^ Furthermore, weight loss has been shown to vary according to initial body weight,^28^ and a clinically relevant weight loss is achieved across baseline BMI categories,^38^ supporting that weight‐adjusted dosing is not necessary.

A previous population pharmacokinetic analysis of liraglutide dosed up to 3.0 mg also identified body weight as a key covariate affecting exposure in adults with overweight or obesity with or without type 2 diabetes mellitus; sex was additionally identified as a key covariate in this large phase 3 dataset.^26^ The effect of sex was less clear in the present smaller adolescent dataset.

The primary response variable used for the present exposure‐response analysis of liraglutide for weight management was BMI SDS, which is a measure of the number of SDs from the population mean BMI, matched for age and sex.^23^ The exposure‐response relationship with respect to change from baseline in BMI SDS was successfully described by an E_max_ model with baseline BMI SDS and age group as covariates on the placebo response. As the BMI SDS tends to be skewed at higher BMI SDS values,^33^ it was important to evaluate results together with other weight‐related parameters. Supportive exposure‐response analyses were therefore also conducted for the percentage change from baseline in body weight and BMI, change from baseline in waist circumference as well as for the proportion of participants achieving at least 5% weight loss. The exposure‐response relationships for the additional response variables were all successfully described by E_max_ models of exposure‐response.

The difference in the weight‐loss responses observed between adults and adolescents in the placebo group, where adults achieved a greater mean weight loss than adolescents, could have been due to lower adherence to active treatment and/or the diet and exercise program in some adolescents, although linear growth (height increase) in a part of the adolescent population might also have contributed. There were several lower‐than‐expected individual liraglutide concentration measurements in trial 4180 in adolescents, which could also potentially indicate a reduced adherence to the trial medication for some trial participants. Low liraglutide concentrations were also observed in four children in trial 4181, although the reason for this was unknown.^22^ Lower adherence in adolescents compared with adults is also a significant concern in diabetes management.^39^ Clinically, there is an ongoing focus to reinforce adherence to both medication and behavioural strategies such as diet and exercise in both diabetes and obesity management.^39^, ^40^, ^41^

In contrast, for adolescents with the highest liraglutide exposures, there was a tendency for weight‐loss responses being comparable to the adult responses. Although the tendency for a steeper exposure‐response in adolescents was not significant in this small data set, the exposure estimates in trial 4180 were based on sparse pharmacokinetic sampling over a long time period, and the estimates could be influenced by samples obtained during periods with reduced or varying adherence to treatment. Thus, the individual exposure estimates also could reflect the adherence level.

Limitations of the present exposure‐response analysis include a relatively low number of paediatric participants as well as some variability in the populations included in terms of inclusion criteria and background lifestyle therapy. Trial design differed between the trials and included counselling in healthy nutrition and physical activity for all participants, but for the adult participants in trials 1839 and 1922 a calorie‐restricted diet was also included. Moreover, the reduced adherence to treatment in adolescents compared with adults as speculated above could not be confirmed in practice. In the exposure‐response analysis, the value for a 19‐year‐old female or male was used as the adult value for the BMI SDS response variable,^33^ assuming that these growth charts would be representative also for older adults. Strengths of the analysis include an overall high rate of completion among participants across trials. Long‐term exposure data were available through sparse pharmacokinetic sampling, and the availability of data in participants receiving placebo allowed for the described exposure‐response analyses. Finally, the availability of large datasets in adults allowed for prior model development, joint analyses and outcome comparisons.

In summary, the population pharmacokinetic analysis indicated similar exposures in adolescents and adults, with similar body weight ranges between the studied adolescents and adults. Body weight was identified as the most important covariate affecting exposure. An exposure‐response relationship with a larger response with increasing liraglutide exposure was established for BMI SDS change from baseline as well as the percentage change from baseline in body weight and BMI, change from baseline in waist circumference, and for the proportion of participants with at least 5% weight loss. While a lower mean response was observed in adolescents compared to adults, consistent with the placebo response across the exposure range, the added benefits of increasing liraglutide exposures were similar in adolescent and adults with overweight or obesity.

## CONFLICT OF INTEREST

KCCP, PMH, DH and NR are employees of Novo Nordisk and KCCP, PMH and DH own stocks in Novo Nordisk. LDM has received grant support paid to the University at Buffalo from AstraZeneca and Novo Nordisk and fees for serving as a healthcare professional consultant from Novo Nordisk.

## Supporting information


**Appendix S1** Supporting information
