# Supplementary material for: Liraglutide pharmacokinetics and exposure‐response in adolescents with obesity
Source: Pediatr Obes. 2021 May 7;16(10):e12799. doi: 10.1111/ijpo.12799 (PMC8519033; doi:10.1111/ijpo.12799)
Supplement: Supplementary file 1 — Appendix S1 Supporting information [file IJPO-16-e12799-s001.docx]

**Online Supporting Information**

**Liraglutide pharmacokinetics and exposure-response in adolescents with obesity**

Kristin C. Carlsson Petri^1^, Paula M. Hale^2^, Dan Hesse^3^, Naveen Rathor^4^, Lucy D. Mastrandrea^5^

*^1^Department of Quantitative Clinical Pharmacology, Novo Nordisk A/S, Søborg, Denmark*

*^2^Clinical Development, Medical & Regulatory Affairs, Novo Nordisk Inc, Plainsboro, New Jersey*

*^3^Department of Medical & Science - Obesity and Metabolism, Novo Nordisk A/S, Søborg, Denmark*

*^4^Department of Global Medical Affairs, Novo Nordisk A/S, Søborg, Denmark*

*^5^Jacobs School of Medicine and Biomedical Sciences, Division of Pediatric Endocrinology/Diabetes, University at Buffalo, Buffalo, New York*

**Correspondence**

Kristin C. Carlsson Petri, PhD, Modelling specialist, Novo Nordisk A/S, Vandtårnsvej 108, 2860 Søborg, Denmark

E-mail: [kristinpetri.pk@gmail.com](mailto:kristinpetri.pk@gmail.com)

**List of tables**

[**Table S1** Parameter estimates from the base pharmacokinetic model 3](#_Toc63857484)

**List of figures**

[**Figure S1** Diagnostic plots for the final population pharmacokinetic model 4](#_Toc61946657)

[**Figure S2** Graphical analysis of PK and demographic data 5](#_Toc61946658)

[**Figure S3** Box-plots of distribution of baseline body weights (left) and BMI SDS values (right) for adolescents compared to adults 6](#_Toc61946659)

[**Figure S4** Liraglutide exposure in adults and adolescents with doses adjusted to 3.0 mg liraglutide 7](#_Toc61946660)

[**Figure S5** Estimated average liraglutide exposure in trial 4180, by Tanner stage without (top) and with (bottom) adjustment for body weight and dose 8](#_Toc61946661)

**Data cleaning by trial in the population pharmacokinetic (PK) analysis**

**Trial 3630:** 339 observations in 29 adults, no samples below the lower limit of quantification (LLOQ).

**Trial 3967:** 129 observations in 13 adolescents, no samples below LLOQ. 1 sample from 1 individual excluded due to missing PK data.

**Trial 4181:** 146 observations in 13 children, 9 samples below LLOQ. Data for 1 individual with only 1 PK sample (non-completer) and 1 with 12 samples (problems with the injection/pen) were excluded.

# Tables

**Table S1** Parameter estimates from the base pharmacokinetic model

| **Parameter** | **Parameter symbol (unit)** | **Estimate** | **95% CI lower bound** | **95% CI upper bound** | **RSE (%)** | **IIV (%CV)** | **Shrinkage (%)** |
| --- | --- | --- | --- | --- | --- | --- | --- |
| **Absorption rate constant** | KA (1/h) | 0.0804 | Fixed | Fixed | Fixed | NA | NA |
| **Apparent clearance** | CL/F (L/h) | 1.07 | 1.01 | 1.12 | 2.7 | 38.3 | 6.5 |
| **Apparent volume of distribution** | V/F (L) | 13.1 | Fixed | Fixed | Fixed | 38 | 13.9 |
| **NA** | Prop. Error | 43.2 | NA | NA | NA | NA | 6.9 |

Abbreviations: CI, confidence interval; CV, coefficient of variation; RSE, relative standard error.

# Figures

**Figure S1** Diagnostic plots for the final population pharmacokinetic model


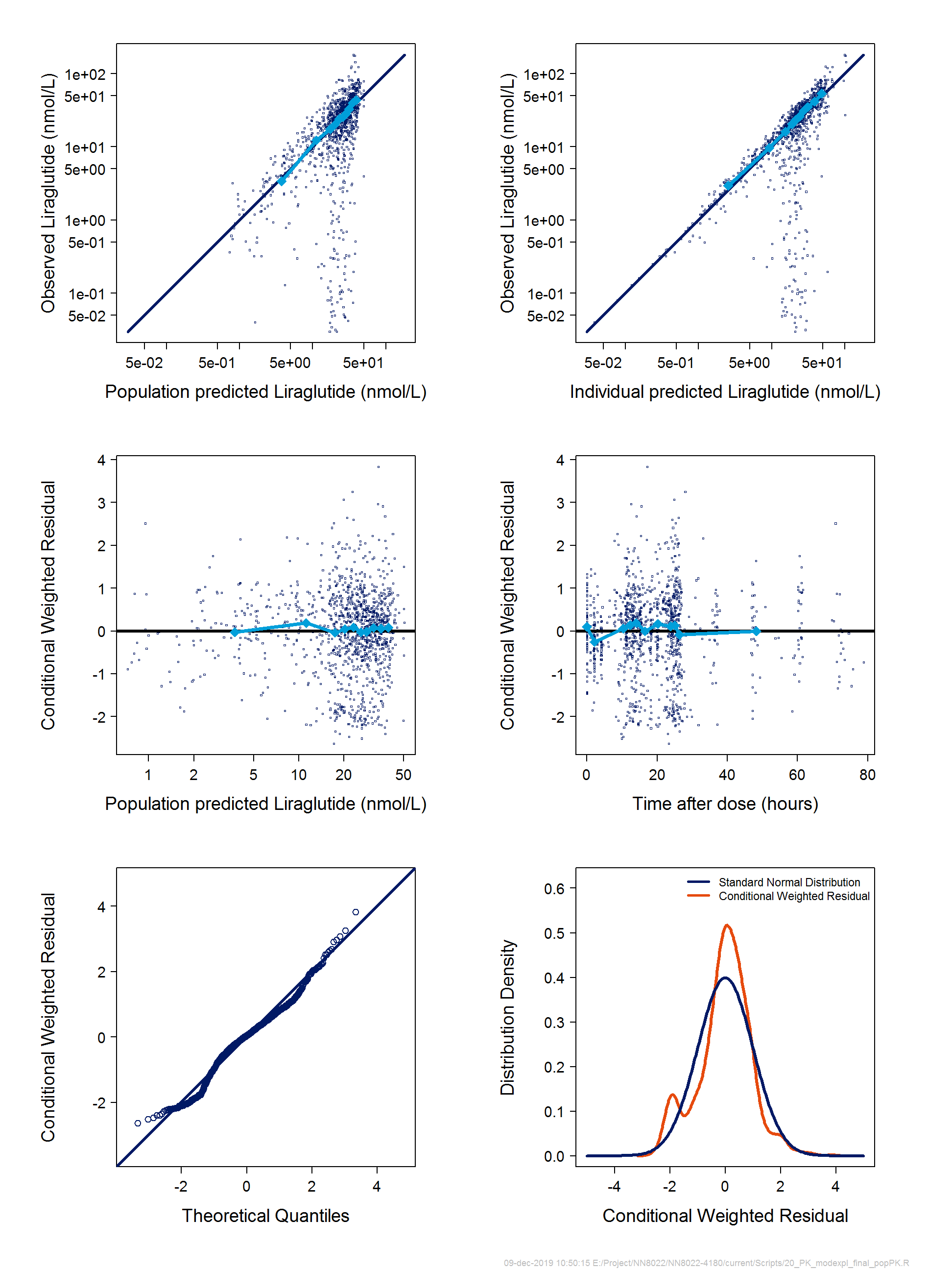


Data are observed concentrations versus population predictions and versus individual predictions, conditional weighted residuals versus population predictions and versus time, QQ-plot of conditional weighted residuals and distribution plot of conditional weighted residuals. Data are included from trials 4180, 3967, 3630 and 4181.

All pharmacokinetic samples were taken when the trial participants were expected to be receiving steady state concentrations of the given dose. Thus, the very low concentrations that are measured in some instances could be due to lack of adherence to trial medication. The second small peak in the conditional weighted residual distribution density (bottom right panel), is caused by these low concentrations.

**Figure S2** Graphical analysis of PK and demographic data


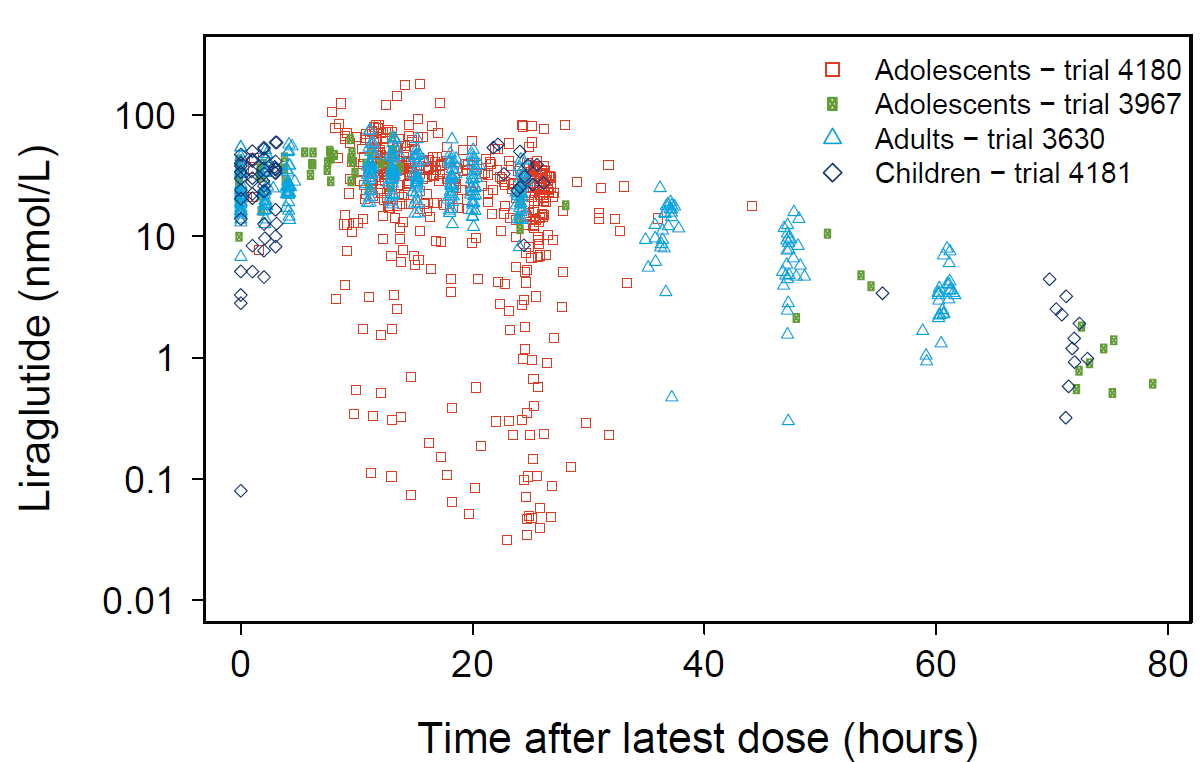


Data are included from trials 4180, 3967, 3630 and 4181.

**Figure S3** Box-plots of distribution of baseline body weights (left) and BMI SDS values (right) for adolescents compared to adults

| 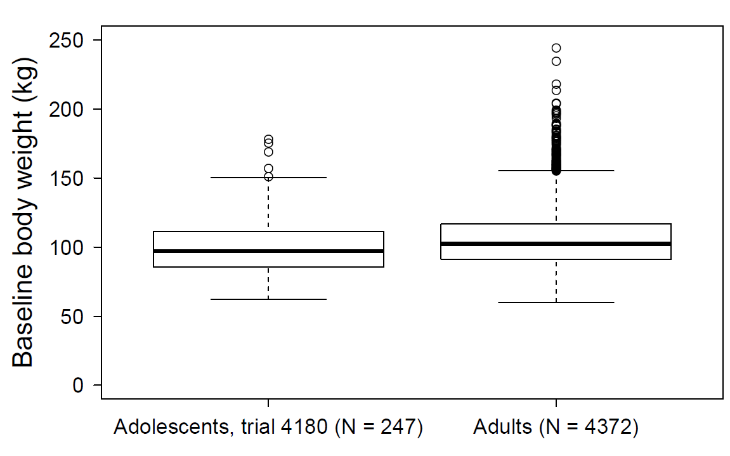 | 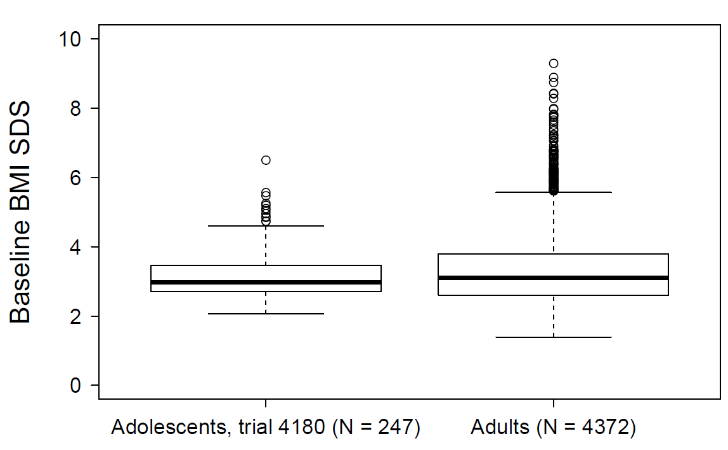 |
| --- | --- |

Abbreviations: BMI SDS, body mass index standard deviation score; N, number of participants.

Boxes represent the 25^th^, 50^th^ and 75^th^ percentiles. Whiskers represent the 5^th^ and the 95^th^ percentiles. Circles are outliers. Data are included from trials 4180, 1807, 1839 and 1922.

BMI SDS was calculated for both adolescents and adults using the WHO 2007 growth reference (https://www.who.int/growthref/en/). For adults, the data for 19-year-olds were used. According to the WHO 2007 algorithm, adjustments are made for high values of BMI SDS. To support the validity of the comparison of BMI SDS in the two age groups, the comparable distributions of baseline body weight and BMI SDS between the two groups are shown above.

**Figure S4** Liraglutide exposure in adults and adolescents with doses adjusted to 3.0 mg liraglutide


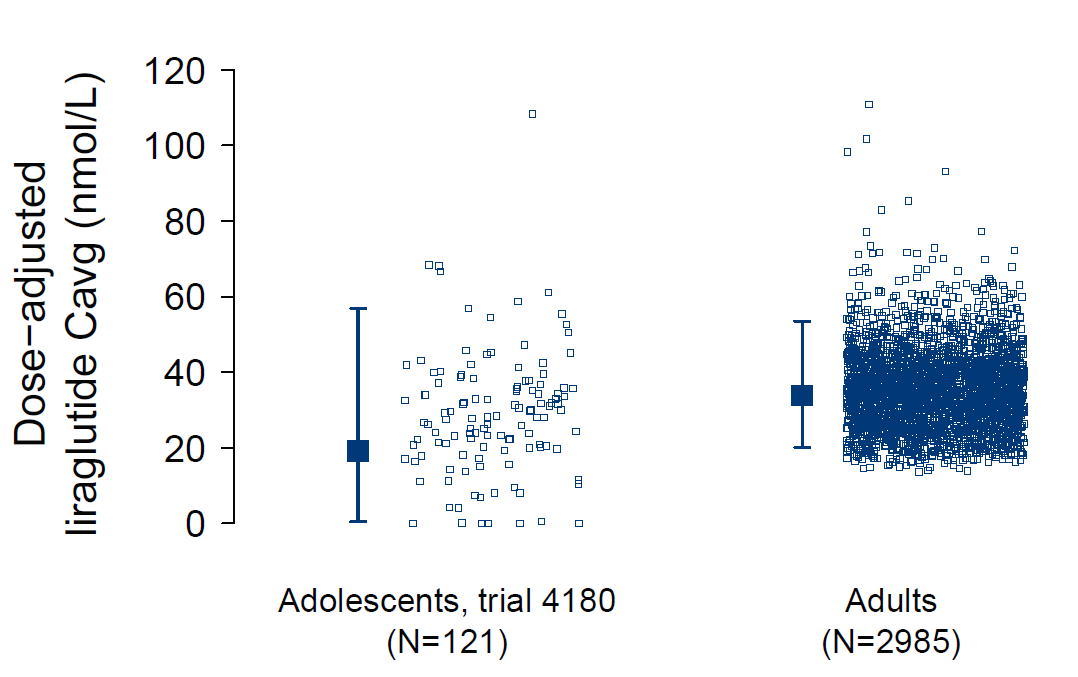


Abbreviations: C_avg_, average liraglutide concentration; N, number of participants; PK, pharmacokinetic.

Data are individual (open symbols) and geometric mean C_avg_ estimates adjusted to the liraglutide 3.0 mg dose with 90% range (closed symbols with error bars) from the final PK model for each trial. Data are included from trials 4180, 1807, 1839 and 1922. In trial 4180, below lower limit of quantification data were included and data cleaning was less strict compared to trials 1807, 1839 and 1922.

**Figure S5** Estimated average liraglutide exposure in trial 4180, by Tanner stage without (top) and with (bottom) adjustment for body weight and dose


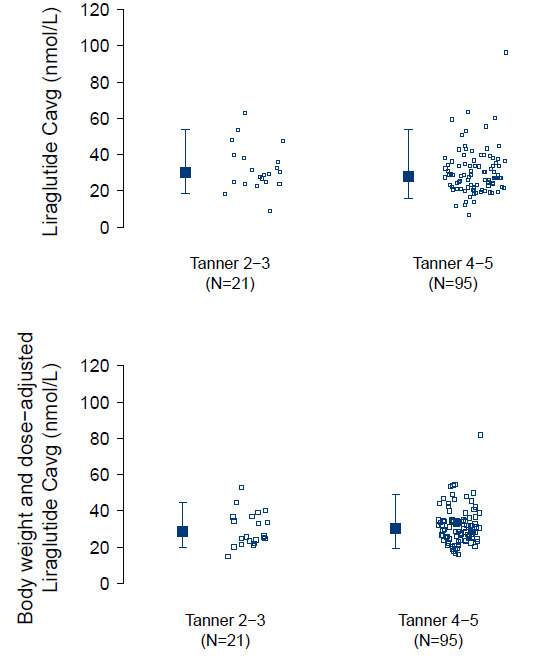


Abbreviations: C_avg_, average liraglutide concentration; CI, confidence interval; N, number of participants; PK, pharmacokinetic.

Top panel: Data are individual (open symbols) and mean C_avg_ estimates with 90% range (closed symbols with error bars) from the final PK model for trial 4180.

Bottom panel: Data are individual (open symbols) and mean C_avg_ estimates adjusted to the liraglutide 3.0 mg dose and body weight with 90% range (closed symbols with error bars) from the final PK model for trial 4180.
